# Scraps

**Published:** 1888-06

**Authors:** 


### SCRAPS

PICKED UP BY THE ASSISTANT-EDITOR.

---

**Accommodating.**—"Take a tablespoonful of this mixture after each meal." "But, doctor, it is so very seldom that I can get a meal," replied the poor patient. "Oh, well, in that case, you can take it before each meal," said the kind-hearted M.D.—*The Medical Age.*

**A Narrow Escape.**—Mérimée, referring to M. Viennet of the French Academy, observed, "We must not speak ill of his tragedies. At the siege of Leipsic he had one in his pocket. A cannon-ball ricocheted against his breast; but the tragedy saved him. The missile had not strength to go beyond the third act."

**Very Mortal.**—A Bristol newspaper one day last month, in giving an account of a recent death of which the cause was at first doubtful, said that the doctor had "stated that death probably resulted from typhoid fever and autopsy." The Editor will perhaps be sorry to hear that there is no record of a patient having recovered from the last-named complication.

**Too Capital a Fee.**—The Ameer of Afghanistan suffered, not long ago, from a boil on his neck, and sought relief at the hands of his Court physician. The latter ordered some sort of a salve to bring the boil to a head. Unfortunately, the application caused the august patient considerable pain, and after a night of torture he sent for his physician and had him decapitated. Evidently the physician to the Ameer does not hold a sinecure.—*The Medical Record.*

**How to Collect Bills for Services.**—Under this heading, Dr. C. Pence, of Spencerville, Ohio, writes to *The Medical Standard:* "The physicians of Spencerville and vicinity have combined for protection against delinquent debtors. The plan adopted is as follows: The physicians make out and exchange lists of patients who are delinquent debtors. All agree to refuse medical aid to such delinquents except for cash. The plan has worked like a charm, and many old debts are being paid. Let physicians elsewhere do likewise."

[Those who think that such a plan would be desirable here should organise it.]

**Bright's Disease.**—John Bright was born in 1811. He made a tour of the Holy Land at the age of 24, but did not desire to purchase it, owing to the existence of a flaw in the title. On his return from the Orient he discovered that what was most needed both in Europe and America, was a good, reliable disease for the use of the better classes. The poor and humble were well supplied, but the rich, the aristocratic and patrician statesmen, corned heads and porkists of the two lands languished for a good, reliable disease that poor people could not obtain. So he began to sit up nights and perfect Bright's disease. He gained the prize at the Paris Exposition, and honourable mention at the great Centennial Celebration at Philadelphia, for "meritorious and effective disease for the better classes." Since that time he has been gratified to notice that the very best people, both in his own land and in this, are handling Bright's disease. It has been kept out of the reach of the poor, and to die from this ailment has been regarded as a proud distinction.—*Journal of Reconstructives,* from the *Boston Globe.*

**Scottish Mediæval Medicine.**—The ninth King of Scots, if we may believe the early chroniclers, rendered his reign memorable by the high honour in which he held physicians. It was in Ireland, where he spent years of exile with his father, says Buchanan, that he became familiar with medicine and its practitioners. When settled on the throne of Scotland, he so inspired the courtiers with his medical tastes that the healing art, particularly surgery, became quite a patrician accomplishment, and for centuries afterwards "nemo esset in Scotia illustriore loco natus, qui vulnerum curationem non teneret." Even down to the sixteenth century surgery, especially military surgery, was a *métier* in which the Scottish aristocracy were proficient. The gallant King James IV. pursued, according to Buchanan, the traditional practice of his race and ancestry with much zeal. "Unum e vetusta gentis consuetudine hausit avide, ut vulnera scientissime tractaret; cujus rei peritia toti Scotorum Nobilitati, ut hominibus in armis assidue viventibus, fuerat priscis temporibus communis."—*The Medical and Surgical Reporter.*

**Special Dispensaries.**—The following extract is taken from an article in *The Hospital.* Referring to matters so thoroughly unknown in this neighbourhood, it will be read with interest, as showing the possibilities of private enterprise.

"FREE DISPENSARY FOR DISEASES OF THE LITTLE TOE,

"—— Street, —— Square.

"In the year 1883, 30,807 persons died of diseases of the little toe. This number, which includes cases of gangrene, pyæmia, phlebitis, and bunion, is exclusive of the very numerous deaths from wasting diseases and cancer of the digit. (See *Registrar-General's Report.*)

"According to the above extract, diseases of the little toe have occasioned more deaths in England than diseases of any other organ, and a comparison of the returns for 1883 with those of previous years shows that these diseases are increasing in a ratio greatly out of proportion to the increasing population.

"*Deaths from Diseases of the Little Toe.*

| Year. | | Year. | | Year. | |
|---|---|---|---|---|---|
| 1874 ... ... | 13,846 | 1877 ... ... | 17,908 | 1880 ... ... | 21,915 |
| 1875 ... ... | 15,401 | 1878 ... ... | 19,634 | 1881 ... ... | 20,940 |
| 1876 ... ... | 15,009 | 1879 ... ... | 20,879 | 1882 ... ... | 24,802 |

1883... ... ... ... ... ... **30,807.**

"Besides the above number of fatal cases, there is a still greater number of diseases of the little toe not terminating in death, but which give rise to extreme suffering and misery, especially amongst the poorer classes. *This is the only institution specially devoted to the treatment of diseases of the little toe,* and the fact that in less than two years upwards of 10,560 patients (from all parts of England) have been relieved proves that it is a necessity. The employment of the newly-invented instrument (the little-toe scalpel) is a special feature in the institution, and by the aid of this instrument morbid conditions of the digit can be fully exposed and adequateiy treated. Further aid is required to meet the urgent demands caused by the daily increasing number of out-patients. It is likewise essential that a ward should be speedily opened for the reception of the more acute cases. *At least £1,000 is required to establish a ward containing six beds.* For this purpose the Committee have determined to open a separate subscription list. Those who desire to assist in this particular way will please notify that their subscriptions or donations are to go to the 'Ward Fund.' The benevolent are repectfully informed that the very existence of the institution will be jeopardised unless the funds are considerably augmented.

"JEREMIAH DIDDLER, Secretary."

